# Identification of a ubiquitin-protein ligase MaUPL6 modulating the response to Fusarium wilt in banana

**DOI:** 10.1186/s43897-024-00129-9

**Published:** 2025-02-05

**Authors:** Yaoyao Li, Jingfang Shi, Yile Huo, Xueyi Xie, Qiaosong Yang, Chunhua Hu, Ou Sheng, Fangcheng Bi, Chunyu Li, Ganjun Yi, Wei Wei, Tongxin Dou

**Affiliations:** 1 Institute of Fruit Tree Research, Guangdong Academy of Agricultural Sciences; Key Laboratory of South Subtropical Fruit Biology and Genetic Resource Utilization, Ministry of Agriculture and Rural Affairs; Guangdong Provincial Key Laboratory of Science and Technology Research on Fruit Trees, Guangzhou, Guangdong 510640 China; 2 Guangdong Provincial Key Laboratory for Crop Germplasm Resources Preservation and Utilization, AgroBiological Gene Research Center, Guangdong Academy of Agricultural Sciences, Guangzhou, Guangdong 510640 China; 3 College of Horticulture, South China Agricultural University, Guangzhou, Guangdong 510640 China; 4 School of Life Sciences, Guangzhou University, Guangzhou, Guangdong 510006 China; 5 Laboratory of Lingnan Modern Agriculture Project, Guangzhou, Guangdong 510642 China; 6 Maoming Branch, Guangdong Laboratory for Lingnan Modern Agriculture, Maoming, Guangdong 525000 China

Bananas (*Musa* spp.) are among the most produced, traded, and consumed fruits globally. However, the destructive disease, caused by the soil-borne fungus *Fusarium oxysporum* f. sp. *cubense* Tropical Race 4 (*Foc* TR4), poses a serious threat due to its wide host range and ability to cause extensive banana losses (Zhang et al. 2024). Despite ongoing research, the molecular mechanisms underlying banana resistance to Fusarium wilt remain poorly understood, necessitating further efforts to enhance the resistance of banana cultivars to this devastating pathogen.

Recent breakthroughs have significantly advanced our understanding of the genetic structure and evolutionary dynamics underlying the Fusarium wilt of banana. Pivotal studies have pinpointed the key effector protein of the *Foc* TR4 strain and mapped out the pathogen's signal transduction pathways (Liu et al. 2020; Zhang et al. 2024). However, the molecular mechanisms driving banana's response to Fusarium wilt remain largely unknown. To date, only a handful of resistance- or susceptibility-related genes, such as *MpICE1* (Li et al. 2022), *MaLYK1* (Zhang et al. 2019), *RGA2* (Dale et al. 2017; Li et al. 2024), *MaERF12* and *MaSMG7* (Huang et al. 2024a), *MaATG4B* and *MaATG8F* (Huang et al. 2024b), have been successfully identified. This highlights the urgent need for further research to discover and characterize the critical resistance- or susceptibility-related genes, especially those responsive to the *Foc* TR4 strain, as the existing knowledges are still woefully inadequate.

In previous studies, we identified the bHLH transcription factor MpICE1 as a crucial positive regulator in modulating resistance to Fusarium wilt by enhancing banana's reactive oxygen species (ROS) scavenging system (Li et al. 2022). This study aimed to elucidate the molecular mechanisms underlying MpICE1-mediated responses to Fusarium wilt. We employed a yeast two-hybrid (Y2H) library screening assay using MpICE1 and identified the HECT ubiquitin-protein ligase (UPL) MaUPL6, which is homologous to AtUPL6 in *Arabidopsis* (Fig. 1A). Given the challenges associated with the genetic transformation of banana plants, we utilized a transient silencing approach to investigate the role of MaUPL6 following *Foc* TR4 infection. Specifically, *MaUPL6*-dsRNAs were injected into banana leaves to assess the gene's function in response to Fusarium wilt. The expression level of *MaUPL6* was significantly reduced in dsRNA-treated leaves compared to water-treated controls (Fig. 1B). After *Foc-*TR4 injection, banana leaves treated with *MaUPL6*-dsRNA exhibited smaller necrotic areas than those treated with water (Fig. 1C, D). These results suggest that MaUPL6 negatively regulates Fusarium wilt resistance in bananas.

**Fig. 1:**
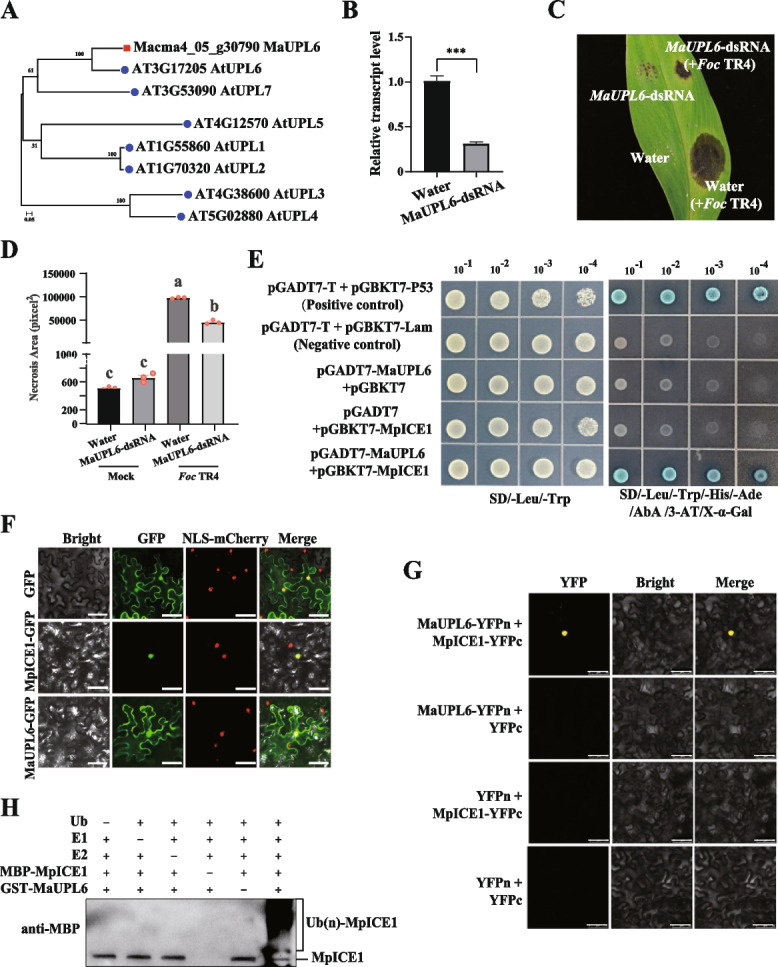
MaUPL6 modulates MpICE1 stability via a ubiquitination pathway, regulating the response to Fusarium wilt in banana. **A** Phylogenetic analysis of the HECT ubiquitin-protein ligase MaUPL6 and the UPL gene family in *Arabidopsis thaliana*. The phylogenetic tree was constructed using the Maximum Likelihood method with MEGA 7.0 software. **B** Expression levels of *MaUPL6* in dsRNA- and water-treated banana leaves determined by qRT-PCR analysis. Data are presented as mean ± S.E. from three biological replicates, with statistical significance determined by a two-tailed Student’s t-test (***P < 0.001). **C** Visual disease symptoms and (**D**) quantification of necrosis area in banana leaves following treatment with *MaUPL6*-dsRNA and water, after a Fusarium wilt inoculation. Necrosis areas were calculated using ImageJ V1.8.0 software. Data are presented as mean ± S.E. from three biological replicates. Statistical significance is indicated by different letters (*P* < 0.05) by Duncan's multiple range test. **E** Yeast two-hybrid (Y2H) assay demonstrating the interaction between MpICE1 and MaUPL6. **F** Subcellular localization of MpICE1 and MaUPL6 in tobacco leaf epidermal cells. Scale bar = 50 μm. **G** Bimolecular fluorescence complementation (BiFC) further confirmed the physical interaction between MpICE1 and MaUPL6. Scale bar = 50 μm. **H** In vitro, a ubiquitination assay showing MpICE1 polyubiquitination by MaUPL6 was detected via immunoblotting with an anti-MBP antibody

Next, we confirmed the interaction between MaUPL6 and MpICE1 by yeast two-hybrid (Y2H) assay (Fig. 1E). Furthermore, the subcellular localization analysis revealed that MpICE1 was exclusively localized to the nucleus, whereas MaUPL6 was present in both the nucleus and the cytoplasm (Fig. 1F), suggesting the potential for interaction between MpICE1 and MaUPL6 within the nucleus. To validate this interaction in vivo, we performed the bimolecular fluorescence complementation (BiFC) assays in *Nicotiana benthamiana* leaves. Strong YFP fluorescence signals were observed across epidermal cells co-expressing MaUPL6 and MpICE1 (Fig. 1G). These observations suggest that MaUPL6 physically interacts with MpICE1 in vivo and in vitro.

Given that E3 ubiquitin ligases are critical enzymes responsible for regulating the proteome through the catalysis of ubiquitination (Langin et al. 2023), we investigated whether MpICE1 could be a substrate for ubiquitination by the E3 ubiquitin ligase MaUPL6. To test this, we purified MBP-MpICE1 and GST-MaUPL6 fusion proteins, respectively, and performed an in vitro ubiquitination assay, which confirmed that recombinant MaUPL6 indeed ubiquitinated MpICE1 in the presence of E1 (ubiquitin-activating enzyme, UBA), E2 (ubiquitin-conjugating enzyme, UBC), and Flag-tagged ubiquitin proteins (Flag-Ub) (Fig. 1H; Fig. S1). Our findings reveal that MaUPL6 ubiquitinates MpICE1, and reduces the Fusarium wilt resistance in banana.

In conclusion, this study offers new insights into the biological role of MaUPL6 as a Fusarium wilt susceptibility-related gene in bananas, providing the first evidence that the ubiquitination system significantly influences the resistance to this disease. However, further research, particularly the *MaUPL6* transgenic lines obtained through the banana transgene technology (Hu et al. 2023), is necessary to substantiate the scientific hypothesis that MaUPL6-mediated ubiquitination of MpICE1 results in its degradation. It is essential to fully understand the precise function of the MaUPL6-MpICE1 module in the banana Fusarium wilt response.

## Supplementary Information

Supplementary Material 1

Supplementary Material 2

## Data Availability

The datasets used during the current study are available from the corresponding author upon reasonable request.
