# Supplementary material for: Identification of a ubiquitin-protein ligase MaUPL6 modulating the response to Fusarium wilt in banana
Source: Mol Hortic. 2025 Feb 5;5:12. doi: 10.1186/s43897-024-00129-9 (PMC11796118; doi:10.1186/s43897-024-00129-9)
Supplement: Supplementary file 1 — Supplementary Material 1 [file 43897_2024_129_MOESM1_ESM.docx]

**Materials and Methods**

**dsRNA-Mediated Gene Silencing in Banana**

The dsRNA-mediated gene silencing assays in banana were conducted following the protocol (Huang et al. 2024). In brief, the *MaUPL6* gene fragment was inserted into the pClone007 vector. Subsequently, *MaUPL6*-dsRNA was synthesized using the T7 RNAi transcription kit. The banana leaves were detached and uniformly wounded using a pipette tip. The wounds were treated with 10 μL of *MaUPL6*-dsRNA at a concentration of 500 ng/μL, combined with 0.02% Silwet L-77. Control sites on the same leaves were treated with 10 µL of water containing 0.02% Silwet L-77. After treatment, the leaves were allowed to dry for approximately 1 hour before being inoculated with mycelia from a 6-day-old *Foc* TR4 strain II5 (NRRL#54006). Fungal lesion sizes were subsequently quantified using ImageJ V1.8.0 software.

**RNA extraction and qRT-PCR analysis**

Total RNA was extracted from Cavendish banana leaves using a plant RNA extraction kit (Code No. 9767, TaKaRa, Dalian, China). Subsequently, cDNA synthesis was carried out with 1 µg of total RNA using the PrimeScript RT Reagent Kit (TaKaRa, Dalian, China). Quantitative real-time polymerase chain reaction (qRT-PCR) was performed to evaluate the transcription levels of *MaUPL6*, employing the qPCR SYBR Green Master Mix (Toyobo). *MaACT1* was used as the reference gene, as detailed by Zhao et al. (2013). The primers used for the qRT-PCR assay are listed in Table S1.

**Yeast Two-Hybrid (Y2H) Assays**

In the Yeast Two-Hybrid (Y2H) assays, the full-length coding sequences of *MpICE1* and *MaUPL6* were cloned into the pGBKT7 and pGADT7 vectors, respectively. These constructs were co-transformed into Y2H Gold yeast strains and cultured at 28°C on SD/-Leu/-Trp medium. The yeast cells were subsequently transferred to SD/-Ade-His-Leu-Trp selection medium containing 10 mM 3-amino-1,2,4-triazole (3-AT), AbA (Aureobasidin A), and α-galactosidase (X-α-gal). Plates were incubated for 2-3 days at 28°C. Interactions were assessed based on growth and α-gal activity, following the yeast protocol manual (Clontech). All primer pairs are listed in Table S1.

**Subcellular localization analysis**

In the subcellular localization analysis assay, the coding sequences of *MpICE1* and *MaUPL6*, excluding the terminator, were cloned into the pBE-GFP vector. These plasmids were then transiently introduced into *Nicotiana benthamiana* leaves through *Agrobacterium tumefaciens* strain EHA105 infiltration. Fluorescence signals of GFP and mCherry were observed two days post-infiltration using a fluorescence microscope (Zeiss Axio Imager D2) equipped with GFP (excitation: 470/40 nm; emission: 525/50 nm) and mCherry (excitation: 550/25 nm; emission: 628/40 nm) filters. The primer sequences used are listed in Table S1.

**Bimolecular fluorescence complementation assays**

In the bimolecular fluorescence complementation (BiFC) assay, the full-length coding sequences of *MpICE1* and *MaUPL6*, excluding stop codons, were cloned into the pCAMBIA1300-N and pCAMBIA1300-C vectors, respectively. These fusion vectors, along with the empty pCAMBIA1300-N and pCAMBIA1300-C vectors, were introduced into *Agrobacterium* strain GV3101. The *Agrobacterium* was then co-infiltrated into *Nicotiana benthamiana* leaves in various combinations. After 48 hours, yellow fluorescence signals in the infiltrated leaves were visualized using confocal microscopy. The primer sequences used are detailed in Table S1.

**In vitro ubiquitination assay**

The ubiquitination assay was conducted following the protocol as described previously (Shan et al. 2020). In summary, the coding sequences of *MaUPL6* and *MpICE1* were inserted into the GST-tagged pGEX-4T and MBP-tagged pMAL-c2X vectors, respectively. These plasmids were then transformed into BM Rosetta (DE3) cells to induce protein expression. The recombinant MBP-MpICE1 was incubated with ubiquitin, E1, E2, and recombinant GST-MaUPL6, and the reaction products were analyzed via immunoblotting using an anti-MBP antibody. Detailed primer pairs are listed in Table S1.

**REFERENCES**

Huang HQ, Liu SW, Huo YL, Tian YZ, Liu YS, Yi GJ, Li CY. 2024. MaSMG7-Mediated Degradation of MaERF12 Facilitates Fusarium oxysporum f. sp. cubense Tropical Race 4 Infection in Musa acuminata. Int J Mol Sci. 2024; 25:3420.

Shan W, Kuang JF, Wei W, Fan ZQ, Deng W, Li ZG, Bouzayen M, Pirrello J, Lu WJ, Chen JY. MaXB3 modulates MaNAC2, MaACS1 and MaACO1 stability to repress ethylene biosynthesis during banana fruit ripening. Plant Physiol. 2020; 184:1153–1171.

Zhao ML, Wang JN, Shan W, Fan JG, Kuang JF, Wu, KQ, Li XP, Chen WX, He FY, Chen JY, Lu WJ. nduction of jasmonate signalling regulators MaMYc2s and their physical interactions with MaIce1 in methyl jasmonate-induced chilling tolerance in banana fruit. Plant Cell Environ. 2013; 36:30–51.


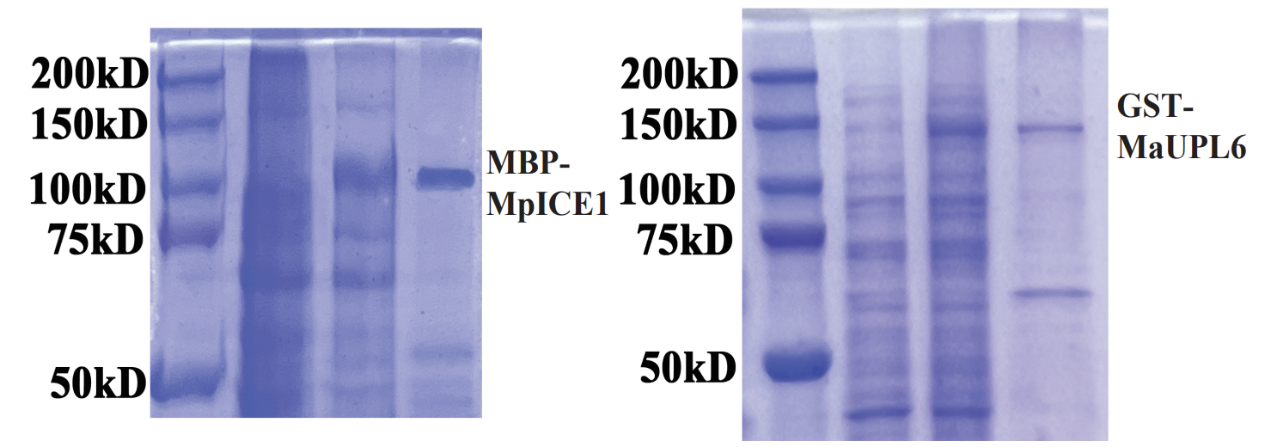


**Fig. S1 The MBP-MpICE1 and GST-MaUPL6 fusion proteins were subjected to analysis via SDS-PAGE (sodium dodecyl sulfate-polyacrylamide gel electrophoresis).**
